# The Purinergic Nature of Pseudoxanthoma Elasticum

**DOI:** 10.3390/biology13020074

**Published:** 2024-01-26

**Authors:** Gilles Kauffenstein, Ludovic Martin, Olivier Le Saux

**Affiliations:** 1 UMR INSERM 1260, Regenerative Nanomedicine, University of Strasbourg, 67084 Strasbourg, France; 2 PXE Consultation Center, MAGEC Nord Reference Center for Rare Skin Diseases, Angers University Hospital, 49000 Angers, France; lumartin@chu-angers.fr; 3 MITOVASC—UMR CNRS 6015 INSERM 1083, University of Angers, 49000 Angers, France; 4 Department of Cell and Molecular Biology, John A. Burns School of Medicine, University of Hawaii at Manoa, Honolulu, HI 96822, USA

**Keywords:** PXE, GACI, calcification, purinergic signaling, adenosine, ATP

## Abstract

**Simple Summary:**

Pseudoxanthoma Elasticum (PXE) is an inherited disease that manifests in abnormal elastic fiber calcification in the eyes, the skin and the blood vessels. The gene responsible, called *ABCC6*, was identified in 2000 but it took nearly 15 years to understand how it works and how this gene’s function relates to elastic fiber calcification. In essence, ABCC6 functions with at least two other enzymes (ENPP1 and CD73) to produce two inhibitors of calcification called pyrophosphate (PPi) and adenosine. In the absence of ABCC6, there is less PPi and adenosine produced, which leads to abnormal calcification in PXE patients. Remarkably, when ENPP1 or CD73 are mutated and non-functional this leads to other inherited diseases called General Arterial Calcification of Infancy (GACI) and Calcification of Joints and Arteries (CALJA), with overlapping symptoms. The three genes (ABCC6 → ENPP1 → CD73) normally work together to not only produce PPi, to prevent abnormal calcification, but also adenosine, which possesses numerous biological functions via a process called purinergic signaling. Our own work and a review of the scientific literature now indicate that PXE (and also GACI and CALJA) is an authentic “purinergic disease”. In this article, we summarize the manifestations of PXE and review molecular and physiological data showing that PXE is indeed associated with a wide range of purinergic systems. Finally, we speculate on the future prospects regarding purinergic signaling and other aspects of this disease.

**Abstract:**

Pseudoxanthoma Elasticum (PXE) is an inherited disease characterized by elastic fiber calcification in the eyes, the skin and the cardiovascular system. PXE results from mutations in *ABCC6* that encodes an ABC transporter primarily expressed in the liver and kidneys. It took nearly 15 years after identifying the gene to better understand the etiology of PXE. ABCC6 function facilitates the efflux of ATP, which is sequentially hydrolyzed by the ectonucleotidases ENPP1 and CD73 into pyrophosphate (PPi) and adenosine, both inhibitors of calcification. PXE, together with General Arterial Calcification of Infancy (GACI caused by *ENPP1* mutations) as well as Calcification of Joints and Arteries (CALJA caused by *NT5E*/CD73 mutations), forms a disease continuum with overlapping phenotypes and shares steps of the same molecular pathway. The explanation of these phenotypes place ABCC6 as an upstream regulator of a purinergic pathway (ABCC6 → ENPP1 → CD73 → TNAP) that notably inhibits mineralization by maintaining a physiological Pi/PPi ratio in connective tissues. Based on a review of the literature and our recent experimental data, we suggest that PXE (and GACI/CALJA) be considered as an authentic “purinergic disease”. In this article, we recapitulate the pathobiology of PXE and review molecular and physiological data showing that, beyond PPi deficiency and ectopic calcification, PXE is associated with wide and complex alterations of purinergic systems. Finally, we speculate on the future prospects regarding purinergic signaling and other aspects of this disease.

## 1. Introduction

Physiological calcification is a metabolic process normally restricted to bones and teeth and primarily leading to the formation of hydroxyapatite crystals (also referred to as calcification or mineralization herein). Hydroxyapatite forms via the precipitation of calcium and phosphate salts and represents 65–70% of the mass of bone and 70–80% of dentin in teeth. Under normal physiological conditions, calcium and inorganic phosphate (Pi) concentrations are near saturation in most soft tissues, which necessitates strong calcification inhibition [1]. The prevention of calcification in soft tissues is an active mechanism that relies on multiple physiological mineralization inhibitors such as Matrix Gla Protein (MGP), fetuin-A, osteopontin and the inorganic pyrophosphate (PPi) [2]. Many host, environmental and genetic factors that contribute to the regulation of pro- vs. anti-mineralization processes have been identified [3], but there are still important gaps in our mechanistic understanding of pathological calcification. These gaps are critical barriers to developing comprehensive diagnostic and therapeutic strategies against ectopic calcification [4]. Ectopic calcification is seen in common cardiovascular diseases such as atherosclerosis, type 2 diabetes (T2D), chronic kidney disease (CKD), aortic stenosis (AS) and senile degeneration [3]. Abnormal mineralization produces arterial stiffness, valvular dysfunction and heart failure, and represents a significant health concern [5]. There are several types of vascular calcification depending on the nature of the vascular layer affected and the associated risk factors [6]. Intimal calcification affects the endothelial layers of medium/large size elastic arteries and is primarily associated with atherosclerotic plaques. Mönckeberg’s media-sclerosis, or mediacalcosis, affects the media of arteries and is mostly seen in type 2 diabetes or chronic kidney diseases [7].

**Figure 1:**
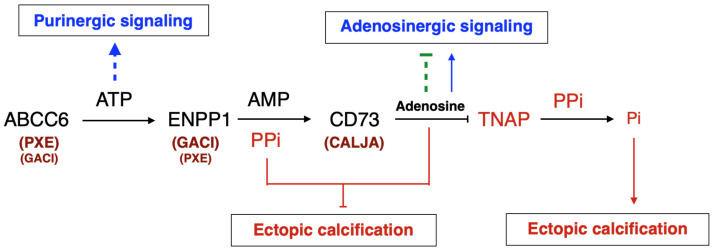
**The ABCC6 pathway influences calcification regulation via extracellular purinergic metabolism**. ABCC6 facilitates the cellular efflux of ATP from liver and other tissues/cells, which is quickly converted to pyrophosphate (PPi), a potent inhibitor of mineralization. Decreased plasma PPi levels cause calcification in PXE and GACI. NT5E activity leads to adenosine production, which affects many biological activities including inflammation and inhibition of TNAP synthesis. TNAP degrades PPi into inorganic phosphate (Pi), an activator of calcification which leads to vascular calcification in CALJA patients.

The identification of disease-causing mutations in *ABCC6* as the cause of pseudoxanthoma elasticum (PXE) [8,9,10] and the recent characterization of its function [11] has provided a wealth of new information on the molecular pathways regulating soft tissue calcification [12]. Indeed, ABCC6 facilitates the cellular efflux of nucleotides, notably ATP, which are sequentially converted at the cellular surface into the calcification inhibitors PPi and adenosine. This conversion is mediated by two membrane-bound nucleotidases, ectonucleotide pyrophosphatase/phosphodiestherase 1 NPP1 (ENPP1) and the ecto-5′-nucleotidase (CD73, encoded by *NT5E*) [11,13,14,15], respectively. ABCC6 deficiency mainly causes PXE (MIM#264800) [8,9,16], whereas defective ENPP1 (the key enzyme for PPi production) leads to generalized arterial calcification of infancy (GACI, MIM#208000). Remarkably, some cases of GACI are caused by *ABCC6* mutations (MIM#614473) while *ENPP1* variants can be associated with PXE [17,18]. This duality highlights the phenotypic overlap between these two diseases, with GACI being a very severe form of PXE. Calcification of Joints and Arteries (CALJA, MIM#211800) is another related pathology due to mutations in *NT5E* (encoding CD73) [14]. The lack of CD73 indirectly leads to enhanced PPi degradation [14,15] from reduced adenosine signaling and activation of the tissue-nonspecific alkaline phosphatase (TNAP/*ALPL*) [15,19]. PXE, GACI and CALJA form a disease continuum with overlapping calcification phenotypes and clinical features with distinct yet related molecular mechanisms. The explanation of these phenotypes places ABCC6 as an upstream regulator of a purinergic pathway that notably inhibits mineralization by maintaining a physiological Pi/PPi ratio in connective tissues (Figure 1).

In this review, we present arguments that ABCC6 is a purinergic signaling regulator, making PXE, and by extension GACI and CALJA, an authentic purinergic pathology. We discuss how ABCC6 was linked to ATP efflux and PPi generation but also recent evidence that ABCC6 dysfunction leads to a systemic imbalance in circulating nucleotide levels and ectonucleotidase expression and activity [20]. Because ABCC6, ENPP1 and CD73 are functionally related, we propose that in addition to a PPi deficit, altered purinergic signaling also contributes to PXE manifestations.

## 2. The Genetics of PXE

### 2.1. A Brief History

PXE is a rare disease, with an estimated prevalence of about 1:50,000 in populations of Caucasian descent [21], although this number varies between population groups, in part due to founder effects [22,23,24,25]. Females are more commonly affected than males with a ~2:1 ratio. The first clinical manifestations are generally diagnosed during the second decade of life [26]. The first description of the clinical signs of PXE appeared in the literature nearly 150 years ago [26,27,28]. It was Darier [27] who identified these symptoms as a clinical entity, which he named “pseudoxanthome élastique”. This term remains largely used to this day as pseudoxanthoma elasticum [29]. Angioid streaks in the retina were first described by Doyne in 1889. Two ophthalmologists, Gröenblad and Strandberg, defined the association between angioid streaks and PXE in 1929 and used the term Gröenblad–Strandberg syndrome, which is now rarely used [28,29]. Although many publications were published in the intervening decades, mostly clinical and descriptive studies, the main turns of events came with the identification of the PXE gene in 2000 [8,9,10] and with the generation of knockout mice in 2005 [30,31].

### 2.2. Inheritance

PXE first appeared to be inherited as both autosomal recessive (AR) and autosomal dominant (AD) forms. The first attempt at defining the mode of transmission for PXE was made by Berlyne and co-workers [32]. A dual mode of inheritance was later suggested by Pope [33], generalizing the notion of different subtypes of PXE with two dominant and two recessive forms. In 1988, Neldner proposed a revised classification, arguing that the various subtypes in fact corresponded to different stages of an age-dependent phenotype and he concluded that PXE was primarily inherited (97%) as AR [34]. Despite some dissonant publications in the following decade, researchers agreed that only 2 forms of PXE existed, a primarily recessively inherited disorder and a minor dominant form with incomplete penetrance [35]. The identification of the PXE gene in 2000 [8,9] mostly settled the question of the inheritance. The generation of PXE knockout mouse models provided the “final nail in the coffin” of AD inheritance [30,31] and a publication by Dr. A. Bergen closed the debate declaring that “(these) *findings mark the end of the autosomal dominant PXE segregation myth*” [36].

With the most recent advances in molecular characterizations, we now understand that PXE, GACI and also CALJA form a spectrum of similar AR diseases with overlapping calcification phenotypes but with vastly different prevalence, PXE being the most common of the three.

### 2.3. The PXE Gene [21]

Using a systematic approach in a pre-genome sequence era, Struk et al. and van Soest and colleagues independently reported a significant linkage of the PXE phenotype to the chromosome 16p13.1 region [37,38], and screening of candidate genes led to the identification of disease-causing mutations in the *ABCC6* gene that was then referred to as *MRP6* in the old nomenclature [8,9,10]. ABCC6 belongs to the ATP-binding cassettes (ABC) superfamily, the largest family of transmembrane proteins. These proteins bind and hydrolyze [28,29] ATP to drive the transport of a wide variety of molecules across cell membranes. Genetic alterations in at least 14 of these genes cause heritable diseases [39,40]. About a third of all these ABC transporter-related diseases are linked to genes from the C subfamily that includes cystic fibrosis (*ABCC7*) [41], and now PXE/GACI with *ABCC6*. Today, more than 400 distinct mutations in *ABCC6* have been reported in patients with classic PXE (https://www.ncbi.nlm.nih.gov/clinvar/?term=abcc6[gene], accessed on 15 November 2023). Disease-causing variants lead to partial or total loss of function of the ABCC6 transporter and include missense and non-sense intronic mutations (causing mis-splicing), insertions and deletions [16,42,43,44,45].

## 3. Disease Manifestations

PXE manifestations affect the skin, the eye and the cardiovascular system with significant phenotypic and severity heterogeneity [26,34].

### 3.1. Skin

The manifestations primarily result from elastorrhexis, i.e., the fragmentation and calcification of elastic fibers in the dermis. The skin lesions are generally the first signs of PXE observed during childhood or adolescence and often progress slowly and unpredictably. Therefore, a dermatologist frequently makes the initial diagnosis. The accumulation of abnormal calcified elastic fibers in the mid-dermis produces the skin lesions, which consist of yellowish papules and plaques, and laxity with a loss of elasticity. These lesions can be seen on the face, neck, axilla, antecubital fossa, popliteal fossa, groin and periumbilical area [34]. The diagnosis generally consists of a skin biopsy of an affected area with von Kossa or Alizarin Red S histological staining that will show calcification and disrupted elastic fibers (elastorrhexis) in the mid- and lower dermis and genetic testing [46]. Beyond their cosmetic appearance, these lesions do not cause any pathological complications and cosmetic surgery (skin resection) is currently the only, albeit incomplete and transient, treatment possible.

### 3.2. Eyes

Ocular lesions are another typical characteristic of PXE and are caused by the calcification of elastic fibers in the Bruch’s membrane, resulting in angioid streaks [47]. The majority of PXE patients will eventually develop ocular manifestations. Doyne was the first to describe these ocular streaks in 1889 [48], and Knapp introduced the term “Angioid streaks” for their resemblance to blood vessels [49]. Bilateral angioid streaks are normally seen as linear gray or dark red lines with irregular serrated edges lying beneath normal retinal blood vessels and represent breaks in the Bruch’s membrane [50]. Angioid streaks are completely asymptomatic and can remain undetected late in life until retinal hemorrhages occur. The Bruch’s membrane is not in a true sense a “membrane” but rather a heterogeneous layer separating the choroid from the retina containing elastic fibers and collagen (type I, III and IV). Visual loss is one of the PXE manifestations that impacts the life of patients the most. They have a high risk of developing exudative macular neovascularization secondary to breaks in a calcified and brittle Bruch’s membrane. Photodynamic therapy has been explored but was not found to be useful [51]. Anti-VEGF injections are effective as a palliative therapy [52] and this has become a common symptomatic treatment for PXE patients. However, a recent ophthalmology study revealed an age-dependent accumulation of optic nerve head drusen with high risk of severe inner retinal degeneration in patients with PXE, which seems independent of anti-VEGF injections [53].

### 3.3. Vasculature

In the vasculature, calcification leads to arteriosclerosis, which is frequently doubled with an accelerated atherosclerosis [54]. Common manifestations of PXE include peripheral arterial disease [55] with frequent intermittent claudication, stroke, uncommon gastrointestinal bleeding and occasionally other heterogeneous manifestations such as rete mirabile [56] and carotid hypoplasia [57]. Fragmentation and calcification of the elastic fibers are frequently observed in the media of middle-sized arteries (Mönckeberg’s media sclerosis) [58]. Even if PXE is not life threatening per se, growing evidence suggest that ABCC6 is part of a functional hub of extracellular matrix homeostasis proteins that can lead to major vascular manifestations in the presence of comorbidities [55].

#### 3.3.1. Coronary Artery Disease

Although severe coronary artery disease (CAD) has sporadically been reported in PXE in a few case reports [59,60,61]; myocardial infarction is an uncommon manifestation as most arterial lesions seem to affect mainly peripheral vessels [55,62,63]. The first large systematic characterization of PXE was actually published in 1988 by Dr. Neldner but it did not specifically focus on cardiac manifestations [34]. In this cohort of 100 PXE patients, Neldner reported only one patient with CAD. The in-depth cardiac evaluation of a cohort of 67 French patients in 2013 was remarkable as it was conducted in conjunction with the characterization of an *Abcc6*^−/−^ mouse model [64]. In this cohort, only 3 patients (4%) had verified CAD, and a single subject had an early severe case of CAD. Myocardial ischemia was also prospectively investigated. Treadmill tests were normal in 40 of the patients tested. Single Photon Emission Computed Tomography (SPECT) showed some limited perfusion defects in only two patients out of 27. Overall, the prevalence of CAD in this cohort was similar to that of the European population (7.3%) aged 40 to 70, thus coronary artery diseases cannot be specifically associated with ABCC6 deficiency. Post hoc (10 years) analysis confirmed the long-term absence of significant CAD in this population [65]. Two other studies suggested an increased risk for CAD in carriers of the p.Arg1141X variant [66,67], but a follow-up case-control study with a very large number of participants (66,831) did not find any association between this p.Arg1141X variant (which had a frequency of 0.6%) and ischemic events [68].

#### 3.3.2. Atherosclerosis

Multiple lines of evidence in the literature indicated a pathologic correlation between ABCC6 function and dyslipidemia [69,70,71,72]. To explore this possibility, we conducted a study presenting compelling evidence obtained with both mouse models and PXE patients that indeed ABCC6 deficiency promotes dyslipidemia and atherosclerosis in a haploinsufficient manner, with significant penetrance [54]. Although in this study we did not determine if dyslipidemia and atherosclerosis produced negative outcomes, myocardial ischemia does not seem to be a major complication of PXE.

### 3.4. Heart

If peripheral arterial disease is commonly associated with PXE, cardiac complications with various degrees of severity have only been described in case reports until recently. The actual prevalence of cardiac complications has now been systematically investigated in a few dedicated studies with limited cohorts. Nguyen et al. analyzed cardiac functions in 19 PXE patients and only found minor deviations from accepted values [73]. In another French cohort, cardiac volume, mass, and systolic and diastolic parameters were also within normal range with minor exceptions, suggesting that ABCC6 does not impact baseline heart function [64]. Aside from a few case reports [74,75,76,77], Vanakker et al. reported only one case (2%) of mitral valve prolapse in a cohort of 42 patients [78], which is consistent with the French cohort investigation (4%) [64] and these values do not differ significantly from the general population at ~2.4% [79].

### 3.5. Mice and Dystrophic Cardiac Calcification

In line with the French PXE cohort data, left ventricular (LV) size and function in 2-year-old mice were similar to controls. However, heart weight and cardiomyocyte size were significantly increased in the 24-month-old KO mice [64]. Mungrue et al. have reported an increased infarct size in *Abcc6*^−/−^ mice after ischemia-reperfusion [80], which may also have clinical implications for patients, though myocardial events seem uncommon in PXE as stated above. Interestingly, *Abcc6^−/−^* mice develop a dystrophic cardiac (DCC) phenotype which has long been recognized in certain congenic strains of mice (C3H/HeJ, DBA/2J or 129S1/SvJ). These animals develop soft tissue calcifications sometimes reaching such extensive proportions that it leads to congestive heart failure, especially when experimentally provoked with high fat diets or physical insults [81,82]. A single point mutation in *Abcc6* results in a constitutive decrease of hepatic ABCC6 protein levels and is directly responsible for the DCC phenotype [83,84]. C3H/HeJ mice develop a delayed chronic PXE phenotype as compared to *Abcc6^−/−^* mice, and DBA/2J and 129/SvJ mice also develop soft tissue mineralization [83,85,86,87]. The exact molecular basis of DCC, which depends on PPi and *Abcc6* (not expressed in cardiomyocytes) [88,89,90] remains obscure. Unraveling its understudied pathomechanisms would greatly improve our understanding of PXE.

Over the years, it became apparent that aside from the development of passive ectopic calcification in *Abcc6*^−/−^ mice, physiological challenges such as a high fat diet and physical lesions can lead to an amplified pathophysiological response [54,80,88,91].

Overall, PXE does not appear to be outright associated with specific or frequent cardiac complications. Cardiac events reported in old U.S. PXE cohorts might be in fact due the superimposition of the usual cardiovascular risk factors. Indeed, the presence of cardiac hypertrophy in old *Abcc6*^−/−^ mice and the enhanced susceptibility to atherosclerosis and dystrophic cardiac calcification in animal models suggest that the PXE patients could be susceptible to cardiopathy when comorbidities are present. A recent study adds further support to this possibility [91]. Experimental arterial hypertension was induced by deoxycorticosterone acetate (DOCA-salt) in uni-nephrectomised *Abcc6*^−/−^ mice. DOCA-salt induced an equivalent rise in blood pressure in *Abcc6*^−/−^ mice and control mice. Calcification and significant fibrosis as well as gene expression profiles favoring calcification, fibrosis and extracellular matrix remodeling were seen in both the heart and aorta of *Abcc6* KO animals by comparison to WT mice [91].

## 4. Animal Models

Shortly after the *ABCC6* gene discovery, *Abcc6^−/−^* mouse models were generated by two independent groups in 2005 by homologous recombination leading to the generation of a truncated *Abcc6* transcript [30,31]. These mice breed with a Mendelian distribution of the KO allele and have a normal life span. *Abcc6*^−/−^ mice lack the ABCC6 protein and develop an ectopic calcification phenotype analogous to the human PXE condition. They display spontaneous mineralization in vascular, ocular and renal tissues as well as in testes and remarkably in the capsules of vibrissae (whiskers). The calcification in vibrissae is quantifiable and is a reliable marker of disease progression [92]. These models have become very useful tools to investigate the PXE pathophysiology, specifically abnormal lipid metabolism and atherogenesis [54,69,93], excessive oxidative stress [94,95,96], increased arterial myogenic tone [97], cardiac fibrosis [80,91], dysregulated energy metabolism [20,98] and mitochondrial dysfunction [99,100]. The DCC phenotype of *Abcc6^−/−^* mice has been effectively used as a quick and quantifiable marker of ABCC6 function [45,88,90]. Of note, several of these pathophysiological manifestations involve extracellular nucleotide and nucleoside signaling [101,102,103,104].

## 5. The PXE Pathophysiology Is Both Metabolic and Cellular

Even though PXE was initially considered as an elastic fiber disease [34], the identification of causative mutations in *ABCC6*, a gene primarily expressed in the liver and kidneys, changed that perspective and suggested a metabolic origin to this pathology [105,106]. Several observations and experimentations lent credence to this possibility. PXE affects the vasculature, the skin and the eyes whereas *ABCC6* expression is limited to the liver and kidneys. The first experiments to address the “metabolic” hypothesis showed that serum derived from patients altered elastic fiber formation from normal fibroblasts in vitro [107]. This was followed by the demonstration that serum from *Abcc6^−/−^* mice had a reduced ability to prevent calcification in vitro [108]. It was, however, the allogenic transplant of whisker tissues and the creation of parabiotic pairing of *Abcc6^−/−^* animals sharing circulation with control animals that provided the most compelling evidence that a circulating anti-calcifying factor regulated by ABCC6 caused the mineralization phenotype of PXE [109,110]. The identification of PPi as the circulating anti-calcifying factor has validated the notion of the systemic nature of the PXE pathophysiology [11].

An alternate cellular-based hypothesis was also proposed early on, giving affected tissue/resident cells such as fibroblasts or smooth muscle cells a major role in the development of the pathology. Because the detection of *ABCC6* mRNA or its encoded protein was difficult mostly due to antibody efficacy/specificity and very low mRNA expression levels, this hypothesis had few proponents. Most data in support of the cellular hypothesis derived from cultured dermal fibroblasts, which play a prominent role in the homeostasis of the extracellular matrix (ECM) and its calcification [111]. Note that dermal fibroblasts are the main human cell type that could be readily derived from PXE patients’ skin biopsies. Remarkably, many studies showed cellular and ECM characteristics specific to PXE fibroblasts, which were proposed as an explanation for the dermal manifestations of the disease. These characteristics include enhanced synthesis of elastin and proteoglycan [112], raised MMP-2 degradative potential [113], dysregulated cholesterol metabolism [54,69], increased oxidative stress [96], altered mitochondrial structure and function [99] and enhanced cellular senescence [114].

## 6. The Search for the Substrate(s)

### 6.1. A Restricted Substrate Specificity

Following the discovery of the PXE gene in 2000, significant milestones in PXE research quickly followed. After the identification of *ABCC6* as the causative gene, Ilias et al., and Belinsky et al., used classic inverted vesicle methodology to demonstrate that ABCC6 was a genuine efflux pump able to transport measurable substrates across the plasma membrane such as the glutathione conjugate of N-ethylmaleimide (NEM-GS), leukotriene C4 (LTC4) or the synthetic peptide BQ-123 [42,115]. Ilias et al., also reported that benzbromarone and indomethacin were effective inhibitors. These reports notably suggested a defined and probably restricted substrate specificity for ABCC6, but provided no clue as to the endogenous substrate(s).

### 6.2. Vitamin K: Logical but No Joy

The Matrix Gla Protein (MGP) is an important regulator of bone and soft tissue mineralization that requires carboxylation. MGP deficiency causes Keutel syndrome, an extremely rare autosomal recessive disorder that is faithfully replicated in *Mgp*^−/−^ mice [116]. Remarkably, a PXE-like phenotype was described in association with mutations in *GGCX* [117]. This gene encodes an enzyme that catalyzes the post-translational carboxylation of MGP and OC using vitamin K as a co-factor. As PXE patients have low serum levels of vitamin K [118], it was logical to suggest that ABCC6 transports vitamin K and that insufficient carboxylation of MGP was the cause of calcification in PXE [119]. However, three animal-based studies quickly ruled out vitamin K as having any prominent role in PXE [92,120,121]. Vitamin K was later shown not to be effectively transported by ABCC6 [122]. Although vitamin K is not directly implicated in the etiology of PXE, MGP seem to play a role, most likely secondary to the PPi deficit. Indeed, the undercarboxylated MGP form (and lower expression) was reported to be associated with calcified structures, including elastic fibers in both in vitro and in vivo studies [88,123,124,125]. Moreover, an animal study indicated that warfarin exacerbates ectopic calcification in *Abcc6*^−/−^ animals by influencing the post-translational modification of MGP [126].

### 6.3. Adenosine

The main steps that linked ABCC6 dysfunction to altered purinergic signaling are summarized below.

**Figure 2:**
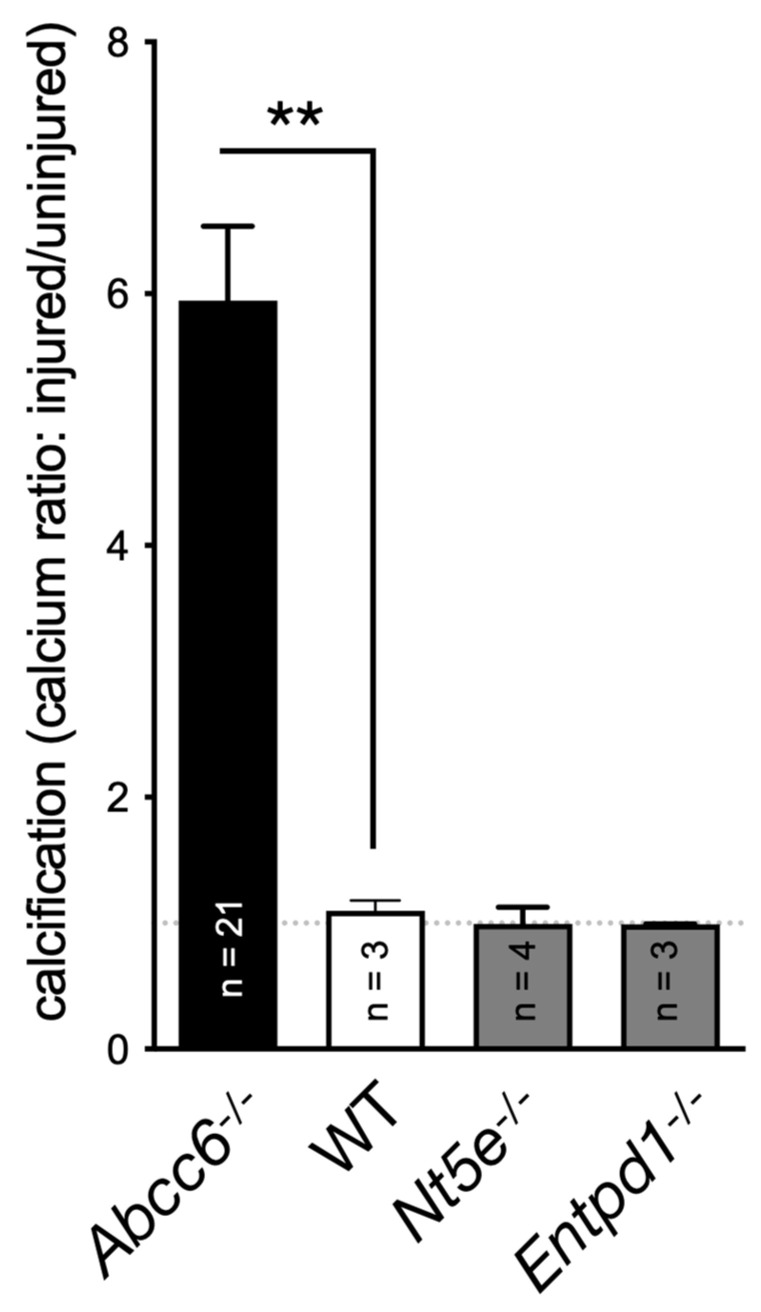
Quantification of the dystrophic cardiac calcification (DCC) phenotype of *Abcc6*^−/−^, *Nt5e*^−/−^ and *Entpd1*^−/−^ mouse models vs. wild type (WT) animals. The calcium content in the murine hearts after cryoinjury, described previously [88], was expressed as a ratio between the injured ventral and uninjured dorsal sections. Results were expressed as mean ± SEM and considered significant for *p* < 0.01 (**).

A key publication in 2011 reported on the rare disease called CALJA. This recessive disease is caused by mutations in *NT5E*, which encodes the ecto-5′-nucleotidase also referred to as CD73 [127]. CD73 catalyzes the hydrolysis of nucleotide monophosphates into their corresponding nucleosides and is the primary enzyme generating extracellular adenosine from AMP [128]. St Hilaire et al. demonstrated that CALJA is associated with defective adenosine signaling, which normally represses TNAP expression via the A_2A_ receptor. In the absence of functional CD73, there is reduced adenosine signaling and the upregulation of TNAP accelerates the hydrolysis of PPi leading to vascular calcifications notably in the lower limb. *Nt5e*^−/−^ mice only partially recapitulate the human phenotype. These animals have an elevated Pi/PPi ratio and develop skeletal hypermineralization at the costochondral junctions and stiffening of the joints, but no vascular calcification or arterial tortuosity [129]. Moreover, we observed that these mice do not develop dystrophic cardiac calcification (Figure 2). The difference could be due to the ½ life of adenosine, which is much longer (8 folds) in mice than in humans [130]. Because of the phenotypic overlap between CALJA and PXE, the authors initially suggested adenosine as a possible ABCC6 substrate [14]. However, Szabo et al., showed that adenosine is not effectively transported by ABCC6 [131].

### 6.4. PPi

One year later, Nitschke et al. added the missing link between ABCC6 and CD73 (Figure 1). Remarkably, this collaborative group of investigators reported that some cases of GACI are only associated with *ABCC6* mutations, while some PXE patients only carry disease-causing *ENPP1* variants [17,132]. This paper revealed an important physiological overlap between PXE and GACI, and more importantly a molecular pathway common to both pathologies.

It is in this context that Jansen and co-workers developed an untargeted metabolomics approach to identify the molecules transported by ABCC6 that could explain its role in the regulation of calcification [11]. The authors found that the expression of ABCC6 leads to a cellular efflux of nucleosides, nucleoside monophosphates and nucleotide sugars, notably ATP, which is rapidly converted into PPi and adenosine at the cellular surface. An in vivo follow-up study confirmed that ABCC6 is responsible for ~60% of plasma PPi levels [13]. Subsequent studies with *Abcc6*^−/−^ and *Enpp1*^−/−^ mice confirmed the pivotal role of PPi production in the control of soft tissue calcification in both PXE and GACI [89,90,133]. The report by Jansen et al. was a major breakthrough in the field of PXE and GACI as it not only paved the way towards credible therapeutic perspectives [19,90,133,134,135] with some already undergoing clinical trials [12], but it was also the first evidence that ABCC6 is positioned upstream of a purinergic signaling pathway: ABCC6 → ENPP1 → CD73 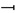 TNAP (Figure 1).

### 6.5. The Question of ATP

Even if the Jansen et al. model is very coherent [11], this work led to the question as to whether ABCC6 could directly transport ATP. Jansen and co-workers measured the uptake of ATP, GTP and UTP into ABCC6-containing vesicles. The results were negative, which suggested that ABCC6 does not transport nucleoside triphosphates directly but could do so indirectly via a different molecular mechanism and/or protein partner. Even though other ABC transporters such as ABCB1 (P-glycoprotein) and ABCC7 (CFTR) have been associated with ATP release [136,137], these stories were controversial at the time [138] but have been revisited more recently [139]. Jansen et al. could not observe increased nucleotide release in a cellular model overexpressing ABCB1, ABCC1, ABCC3, ABCC4, or ABCC5. For now, the exact process by which ABCC6 facilitates ATP cellular efflux remains uncertain. In the future, specific experiments could identify the molecular mechanism implicated in this permeability, but for the time being only speculation is possible. Because pannexin channels, connexin hemichannels, volume regulated anionic channels (VRAC) or ANKH have been associated with the cellular export of nucleotides [140], these could be possible molecular partners. ANKH is also of particular interest. *Ank^−/−^* mice develop progressive ankylosis, with progressive joint calcification and arthritis associated with an imbalance of intra- vs. extracellular PPi. These data were interpreted at that time as an impaired PPi extrusion [141]. In humans, ANKH deficiency leads to bone metabolism dysfunction and craniometaphyseal dysplasia [142,143]. A recent study has shown that ANKH effluxes nucleotide triphosphates from cells, including ATP as well as citrate but not PPi [144,145]. Therefore, depending on the type of cells or tissues, it is possible that membrane channels and/or vesicular transport work in parallel to or instead of ABCC6 to efflux extracellular ATP (and other nucleotides) as precursors of anti-calcifying molecules. The relative importance and tissue distribution of these efflux mechanisms may account for the preponderance of calcification susceptibility of the joints, the vasculature, the skin, or cardiac valves (Table 1).

### 6.6. Plasma PPi Does Not Fully Explain Calcification Susceptibility

The central role of ABCC6 in PXE and GACI is now well established in humans [155] and DCC in animal models [86,156]. However, many aspects of the pathophysiology of ABCC6 dysfunction are still unexplained:

1. If a deficit in PPi production is essential to the etiology of both PXE and GACI and supplementation appears to be a credible therapeutic possibility [12], plasma PPi does not correlate with calcification heterogeneity in humans [18] and mice [85]. Similarly, in a recent report investigating 78 patients and 69 heterozygous, Van Gils et al. found that neither phenotype manifestation/severity nor genotype correlated with plasma PPi [157].
2. The liver expression of ABCC6 is necessary but not sufficient for calcification inhibition [19,88]. The question of how peripheral tissues contribute to calcification inhibition still remains unresolved; however, the recent emergence of inflammation in PXE patients in connection with ABCC6 [158,159,160,161] suggested that the adaptative immune system could be a significant contributor to the calcification phenotype.
3. Adding complexity to the relatively simplistic model shown in Figure 1, dermal fibroblasts of PXE patients also seem to display an impaired ability to generate PPi [162,163] and the crucial role of ANKH in the regulation of local PPi homeostasis [144] shows that in addition to ABCC6 keeping systemic PPi concentrations within the physiological range, extrahepatic PPi production (which cannot be assessed reliably as yet) is also a critical determinant of phenotypic outcome in PXE and GACI.

The clinical relevance of possible modifier genes has been studied [163,164,165,166,167,168,169] but the exact role of modifiers in the pathophysiology of PXE is still not clear and more work needs to be done on this front.

## 7. Altered Ectonucleotidase Activities Associated with ABCC6 Deficiency

Considering the clinical overlap of PXE with GACI and CALJA [127,132,155], it was not surprising that ABCC6 function relates to a cellular efflux of ATP, which is an important precursor to two of the main inhibitors of calcification, PPi and adenosine. However, it is after a series of reports published within a few years of the seminal article of Jansen et al. [11] that the influence of ABCC6 on purinergic-related metabolism came into focus [15,19,162,163]. Miglionico et al., 2014 reported that silencing *ABCC6* in HepG2 cells causes a downregulation of *NT5E* (CD73) and an increase in *ALPL* (TNAP) expression. Because TNAP transcription is repressed via an adenosine A_2A_/cyclic AMP/FOXO1-dependent mechanism [127,170] these data suggested that ABCC6 influences extracellular adenosine levels. As plasma adenosine levels are seemingly normal in *Abcc6^−/−^* mice [20], TNAP dysregulation in PXE is likely restricted to peripheral tissues as suggested by Ziegler et al. [19]. The role of TNAP in the calcification phenotype of PXE is of sufficient importance that its inhibition constitutes one of the most promising treatments to limit ectopic calcification in this disease [12,171].

To better determine the actual influence of ABCC6 deficiency on purinergic-related metabolism, we developed a study to measure circulating ATP and related metabolites and also soluble nucleotidase activities in both PXE patients and *Abcc6^−/−^* mice. This study assessed the expression of genes encoding ectocellular purinergic signaling proteins in the liver with high *Abcc6* expression and the aorta that has little to no expression [20]. Plasma ATP and pyrophosphate levels were markedly reduced in PXE patients and in *Abcc6^−/−^* mice, but surprisingly not adenosine. Moreover, soluble serum CD73 activity was increased in PXE patients and *Abcc6^−/−^* mice as compared to controls, which mirrors the increased levels in C3H/HeJ mice [172], a mouse strain that also harbors a naturally occurring *Abcc6* mutation (cf. chapter 4, Animal Models). Consistent with alterations of purinergic signaling, the expression of genes involved in purine and phosphate transport/metabolism was dramatically modified in the *Abcc6*^−/−^ mouse aorta, but much less so in the liver. Only 8% of the investigated genes were altered in the liver, whereas 38% were changed in the aorta. What is remarkable, is that the most profound changes occurred in the vasculature, a tissue subjected to calcification but with very little *Abcc6* expression, whereas the liver, which has the highest level of expression [106], presents no calcification and significantly less gene expression changes. In support of our observations, altered PPi metabolism was reported in skin fibroblasts isolated from PXE patients [162]. Our study provided the first systemic evidence that ABCC6 function regulates central and distal ectonucleotidase activities and purinergic signaling.

**Figure 3:**
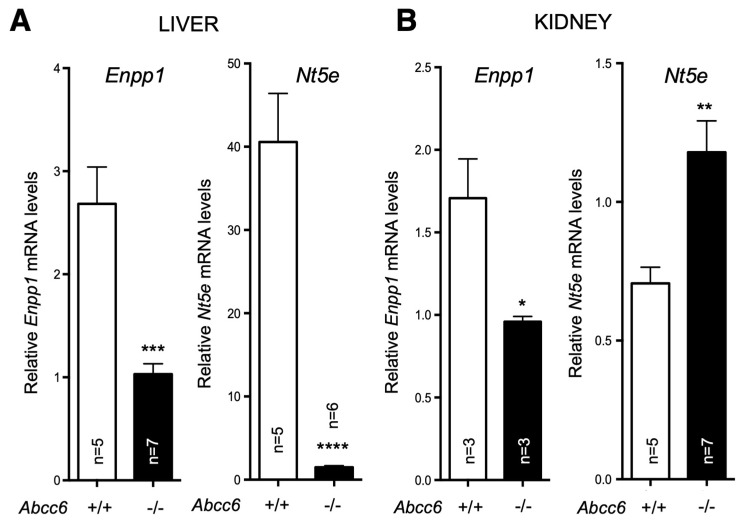
Expression of *Enpp1* and *Nt5e* in liver and kidneys is dysregulated in a tissue-specific manner in *Abcc6*^−/−^ mice. The mRNA levels were determined by Real-Time PCR using specific TaqMan probes to *Enpp1* and *Nt5E*. The data normalized to *Gapdh* are expressed as relative quantities. Results are +/− SEM. * *p* < 0.05, ** *p* < 0.01, *** *p* < 0.001, **** *p* < 0.0001.

In a separate study, we found that both *Enpp1* and *Nt5e (CD73)* genes are indeed differentially dysregulated in the liver of *Abcc6^−/−^* mice [90]. Interestingly, if *Enpp1* seems to be downregulated in the tissues examined, CD73 is upregulated in the vasculature and kidneys of *Abcc6^−/−^* mice (Figure 3) and also shows increased activity in plasma (soluble form of CD73). This further suggested tissue-specific changes of CD73 expression/activity in response to ABCC6 deficiency [15,20,173]. As CD73 is the main enzyme generating extracellular adenosine, one would anticipate auto- and paracrine downstream effects, depending on the types of cells and tissues. Adenosine plays an immunomodulatory role in the liver and contributes to limit inflammation and tissue damage notably during ischemic preconditioning [174,175], in non-alcoholic fatty liver disease and steatohepatitis. Adenosine also promotes fibrosis in the long term [176,177,178,179]. Given the reduced CD73 expression in PXE, low levels of hepatic extracellular adenosine can reasonably be expected, at least in some tissues. If future studies confirm a deficit of adenosine signaling in PXE, this could provide potential therapeutic avenues targeting adenosine receptors or CD73 activity directly. Reduced CD73 activity does not seem to cause obvious liver dysfunction but the work of Miglionico et al. suggested that the consequences likely manifest distally as both Fetuin A and osteopontin expression are also decreased [15]. Furthermore, Ziegler et al. published strong evidence [19] for altered TNAP activity in peripheral tissue, which is consistent with altered adenosine signaling. If the consequence of reduced CD73 expression/activity is obvious with respect to PPi metabolism and ectopic calcification, the consequences of the overexpression of CD73 in renal tissue, vasculature and circulation are less obvious. Adenosine promotes angiogenesis mostly through activation of the A_2A_ and A_2B_ receptors on endothelial cells [180,181]. Because PXE patients experience abnormal choroidal neo-angiogenesis as well as vascular dysplasia (from carotid hypoplasia to carotid rete mirabile [55]), one could reasonably speculate that abnormal adenosine signaling could be responsible for these manifestations. Table 2 summarizes known altered purine metabolism associated with ABCC6 deficiency.

## 8. Impaired Purinergic Signaling—A Connection to Other PXE Manifestations

Nucleotide-based signaling is a primitive but highly conserved system in cells. Purine nucleotides (i.e., ATP, ADP…) and nucleosides (i.e., adenosine) play distinct roles as extracellular transmitters in a wide range of tissues [184,185]. Apart from neurogenic exocytosis, non-lytic release occurs by cellular efflux in response to various stimuli (shear stress, hypoxia, etc.) and are often seen by cells as pro-inflammatory “danger signals” [186]. Adenosine is also considered as vasculoprotective [187]. Once in pericellular space, nucleotides bind to purinergic P2X ion channels or P2Y G-coupled receptors whereas adenosine is recognized by P1 receptors (also known as A_1–3_). Hydrolysis of extracellular nucleotides into adenosine is carried out by ectonucleotidases, which regulate agonist availability, P2 vs. P1 activation and inflammatory status [176,187]. Among the many ectonucleotidases, the ENPP and ENTPD families and CD73 (i.e., NT5E) seem to be the most relevant and act in concert to attenuate ATP signaling and promote adenosine-signaling activation. Disruption of purinergic signaling is involved in many pathophysiological processes and its contribution to vascular remodeling, inflammation, thrombosis and bone mineralization, among others, is well documented [184,188,189,190,191]. Ectonucleotidases, in particular CD39 [192] and CD73, have been shown to contribute to endothelium integrity and prevention of vascular leakage and leucocyte extravasation, cytokine secretion, platelet aggregation and hemostasis. Their role in bone metabolism has been reviewed recently [193].

### 8.1. Immune Cells and Inflammation

We and others have previously reported a significant decrease in plasma purines and changes in ectonucleotidase expression in vivo and in vitro [15,20,54,88,173]. These findings indicated that ABCC6 deficiency not only reduces PPi production in PXE patients and *Abcc6*^−/−^ mice but also dysregulates nucleotide hydrolysis/metabolism. This dysregulation is likely to alter purinergic-related signaling and contribute to non-calcification manifestations in PXE. This assumption is based on the well-established role of extracellular nucleotides as pro-inflammatory molecules [194] vs. the immunosuppressive function of adenosine [191].

Inflammation primarily promotes tissue calcification through macrophage-derived cytokines such as TNF-α, IL-1β and IL-6 [195,196,197]. Although the specific mechanisms involved are not fully understood, an imbalance in the ATP/PPi ratio on the surface of innate immune cells impairs inflammatory and anti-calcification processes, as suggested by Villa-Bellosta et al. [198]. In macrophages, where ABCC6 is not expressed [54], pro-inflammatory polarization affects the expression of *Enpp1* and *Cd39* (i.e., *Entpd1*), leading to a reduction in the ATP/PPi ratio and promoting calcification.

The recent emergence of inflammation as a possible contributor to the progression of calcification in PXE [159,160,161] suggested that immune cells also contribute to calcification regulation. Moreover, the presence of IL-6 in the circulation of mice and humans as reported by Brampton et al. [54] aligns well with the presence in PXE patients of spontaneous erythematous flareup associated with typical PXE skin papules [199]. Indeed, IL-6 is typically produced at sites of acute and chronic inflammation and is also essential for T-cell recruitment and triggers the expression of chemokines, including CCL5, which is also increased in patients [54]. Moreover, positron emission tomography combined with computed tomographic imaging has shown a positive correlation between calcification and inflammation in the skin and arteries of a 42-year-old PXE patient [159]. At this point in time, the connection between ABCC6 function and IL-6 is not clear, however a recent study could provide some clues [200]. A very recent publication by Casemayo et al. showed that *Abcc6* deficiency produced an immune modulation with positive effects on the renal manifestations of rhabdomyolysis [158]. In this study, the authors also demonstrated *Abcc6* expression in CD45+ cells, which independently confirms a direct physiological role for this ABC transporter in immune cells.

**Figure 4:**
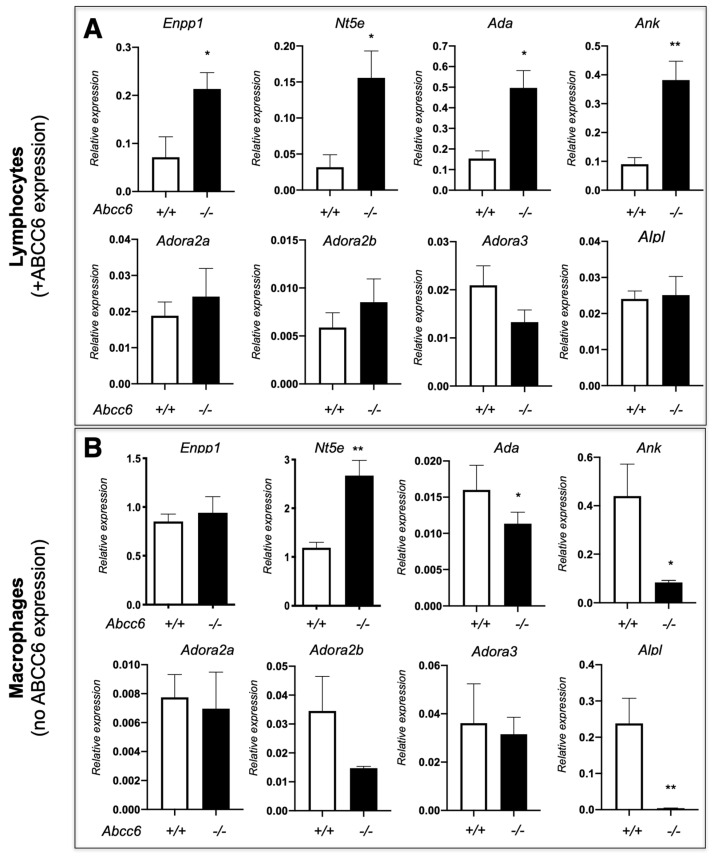
The expression of a few selected purinergic signaling-related genes in lymphocytes (**A**) and macrophages (**B**) isolated from WT (+/+) *Abcc6*^−/−^ (−/−) mice. The expression profile is differentially dysregulated in lymphocytes (*Abcc6* expression) and in non-polarized macrophages (no *Abcc6* expression). The data shows relative gene expression profiles normalized to *Gapdh*. Mouse numbers are 5 vs. 4 in lymphocytes and 6 vs. 6 in macrophages. Results are show +/− SEM. * *p* < 0.05, ** *p* < 0.01.

Our previously published results showed that ABCC6 deficiency modifies the expression of purine metabolism and signaling-related genes in a tissue-specific manner, regardless of *ABCC6* expression status [20]. Consistent with these results, we have observed variable gene expression between lymphocytes and macrophages isolated from *Abcc6*^−/−^ mice. Remarkably, this dysregulation differs in some cases (cf. *Ank*, *Alpl* and *Ada* on Figure 4) indicating that ABCC6 deficiency influences immune cells perhaps through specific autocrine and/or paracrine mechanisms. Interestingly, even in this limited experiment, the lack of ABCC6 seems to affect mostly genes related to PPi (*Enpp1*, *Ank* and *Alpl*), while the CD73-encoding *Nt5e* gene is upregulated in both lymphocytes and macrophages. This suggested that the differential transcriptional regulations of purine metabolism-related genes are systemic, at least in *Abcc6*^−/−^ animals and it is reasonable to suggest that these molecular pathways contribute to and/or aggravate the calcification phenotype. Considering the key role of P1 [191] and P2 [194] receptors in innate immunity and inflammatory processes, we anticipate that dysregulated immune responses and inflammation in PXE result from sub-nominal cytokine patterns linked to altered purinergic signaling. The connection between purinergic alterations, inflammatory processes and their physiological impact(s) as it was shown for other conditions [201] should be a research priority in the coming years.

### 8.2. Vascular Smooth Muscle and Endothelial Cells–CD39

Interestingly, the decrease in plasma ATP that we documented in *Abcc6*^−/−^ mice [20] was not mirrored in *Entpd1*^−/−^ mice lacking NTPDase1/CD39, a major vascular ATPDase [202]. An imbalanced purinergic signaling may be important in disease-affected cells, particularly in vascular smooth muscle and endothelial cells.

Vascular smooth muscle cells (VSMCs) play a significant role in the development of vascular calcifications seen in PXE, GACI, CALJA, CKD and T2D [3]. Under normal conditions, VSMCs exhibit inherent anti-calcifying activity mediated by mineralization inhibitors such as pyrophosphate (PPi) and matrix Gla protein (MGP) [3]. However, in pathological states, VSMCs undergo apoptotic and senescent processes, experience increased oxidative stress and transdifferentiate into osteoblast-like cells [203,204]. As a consequence, they adopt an osteogenic phenotype (which may not systematically translate into macroscopic vascular calcification), and also express genes coding for the transcription factor RUNX-2, osteocalcin, sodium-dependent phosphate co-transporter (PiT-1), bone morphogenetic proteins (BMPs) and tissue-nonspecific alkaline phosphatase (TNAP) among others. The regulation of the Pi/PPi ratio is crucial in VSMC-associated mineralization [205,206]. This ratio depends on the transmembrane transports of Pi and PPi, as well as the transport and abundance of extracellular ATP, along with the activities of cell surface ectonucleotidases [198]. Yet, it remains unclear how the regulation of Pi/PPi ratio in local tissues relates to the systemic plasma PPi levels. This question is reminiscent of the ongoing debate surrounding the systemic versus cellular hypotheses of the PXE pathophysiology.

Under normal conditions, VSMCs primarily express NTPDase1/CD39, responsible for most of the surface ATPDase activity [202], and also ENPP1 [207], the latter being the rate-limiting enzyme for PPi production (along with ATP availability [208]). We observed a significant increase in the expression of genes encoding CD39, CD73 and ENPP1 in the vasculature of *Abcc6*^−/−^ animals. Interestingly, we found no evidence of ABCC6 transporter expression in the wild type VSCMs from micro- or macro-circulation (Kauffenstein et al. Personal communication), although low expression levels could be present in gastrointestinal smooth muscle cells [209]. The possible expression of ABCC6 transporter in smooth muscle cells and its contribution to pathogenic processes, including calcification, certainly needs to be better characterized. However, no clear role of CD39 in soft tissue calcification has been reported and CD39-deficient (*Entpd1*^−/−^) mice did not exhibit significant vibrissae calcification (not shown) or DCC (Figure 2). It is surprising as one might have expected some anti-calcification activity as it competes with ENPP1 for ATP usage, and could possibly have had some influence on PPi production, which seems to depend on the ratio of ENPP1 to ENTPD1 activity [210]. Remarkably, it has recently been shown that hydrolysis of extracellular ATP by VSMCs undergoing chondrocyte-like differentiation generates Pi but not PPi [211], suggesting the loss of pyrophosphate generation and/or an increase in NTPDase activity. While ENPP1 exerts a clear anti-calcifying activity, the role of CD39 in SMC calcification is not obvious and could depend on circumstances.

Therefore, CD39 probably does not have a direct role in pathological calcification in PXE but its contribution to other PXE manifestations is a possibility. Indeed, in addition to the decrease in plasma adenylic nucleotides, we observed a significant increase in the expression of genes encoding CD39, CD73 and ENPP1 in the vasculature of *Abcc6*^−/−^ animals, suggesting alterations in local nucleotide hydrolysis, Pi/PPi ratio and receptor-mediated purinergic signaling via P1- and P2-type receptors. Local concentrations of ATP and ADP in the vasculature can impact inflammatory, thrombotic and other cardiovascular manifestations associated with P2 purinergic receptor activation [189]. Aberrant P2 receptor activation may contribute to the vascular phenotype of PXE and underlie manifestations observed in patients and *Abcc6*^−/−^ mice, such as cardiomyocyte hypertrophy [64], increased resistance arterial tone [97], thrombotic episodes, vascular occlusions [212], increased inflammatory infiltrate after ischemia-reperfusion [80] and fibrosis [213]. Interestingly, the P2_X7_ receptor was also overexpressed in these cardiovascular tissues. This receptor has recently been shown to limit calcification in dystrophic skeletal muscles [214]. In addition, PPi was found to have calcification-independent effects through activation of the P2_X7_ receptor [215]. The role of P2_X7_ in ectopic calcification in the context of ABCC6 deficiency certainly deserves another look.

### 8.3. How Does ABCC6 Function Relates to Cardiac Manifestations?

The adenosine-generating extracellular pathway that ABCC6 regulates (ABCC6 → ENPP1 → CD73 → Adenosine, see Figure 1) is likely to have a significant distal influence towards cardiac function in the presence of comorbidities. A recent report suggested an important role for ENPP1 in post-injury cardiac repair by regulating the purine–pyrimidine balance [216] which opens some interesting perspectives for further exploration of the role of this purinergic pathway. Also, extracellular cAMP hydrolysis into adenosine by ENPP1 reduced cardiomyocyte hypertrophy through A1 adenosine receptors while providing antifibrotic signaling to cardiac fibroblasts via A2 adenosine receptors in a β-adrenergic-dependent model of heart failure [217]. Furthermore, the resolution of inflammation post myocardial infarction involves important changes in extracellular purine metabolism. For instance, the loss of CD73 dramatically alters cardiac function after ischemia/reperfusion, which is followed by a prolonged inflammatory response and enhanced fibrosis [218]. CD73 is highly expressed in T cells infiltrating the ischemic lesions. The resulting adenosine notably signals toward the inhibition of pro-inflammatory (IFN-γ) and pro-fibrotic (IL-17) cytokine production via the A2 receptor. This underlines the importance of adenosine in preventing adverse cardiac remodeling [219]. One should note that the loss of ABCC6 (which facilitates ATP efflux), leads to significant changes in the expression of ENPP1 and CD73 ectonucleotidases in several distal tissues expressing or not expressing *ABCC6* (liver, kidneys) as well as in inflammatory cells (Figure 4). Myocardial mineralization is an under-reported form of ectopic calcification observed in the aging heart and in patients with diabetes, chronic kidney disease, or myocardial injury secondary to ischemia or inflammation [220,221]. Calcification within the heart muscle is a common underlying cause of heart blocks where calcification and fibrosis interrupt the propagation of electrical impulses [222,223]. In pathological circumstances, cardiac fibroblasts adopt an osteoblast cell-like phenotype and contribute directly to the calcification of the heart muscle. ENPP1, which is induced upon cardiac injury, actively contributes to this phenotype [224]. The presence of cardiac hypertrophy in old *Abcc6*^−/−^ mice [64] and the well-characterized dystrophic cardiac calcification in animal models [83,84,88] suggest that the PXE patients could well be susceptible to cardiopathy when comorbidities are present as changes to the cardiac purine metabolism via elements of the ABCC6 → ENPP1 → CD73 → Adenosine pathway could be responsible for the pathological cardiac manifestations reported in the many case reports involving PXE or GACI [225,226,227,228,229,230].

### 8.4. A Compensatory Mechanism?

**Figure 5:**
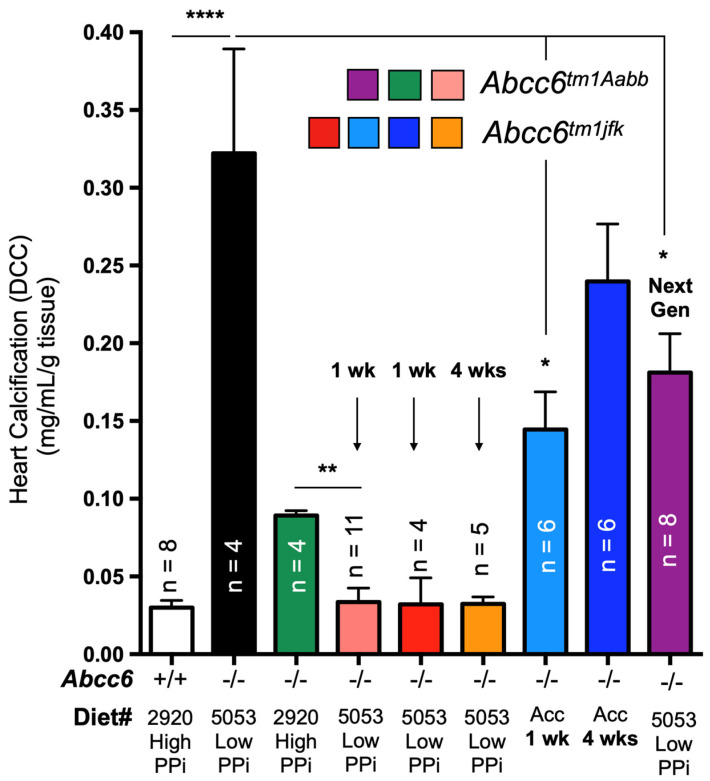
The effect of dietary PPi on the acute dystrophic cardiac calcification (DCC) phenotype of WT (+/+) *Abcc6*^−/−^ (−/−) mice. Mice consumed either a diet with a high (2920) or low (5053) PPi content. Typically, *Abcc6*^−/−^ mice develop limited DCC when fed the high PPi diet as compared to animals fed the low PPi diet. Remarkably, animals born and raised on the high PPi diet for multiple generations failed to return to “normal” DCC susceptibility when exposed to the low PPi chow, even when bred, born and raised for one generation on such a diet. Only the use of an “accelerated diet” [232] restored calcification susceptibility to somewhat similar DCC susceptibility. The level of calcification was measured as total ventricular calcium and normalized to the weight of the tissue. The mice used were approximately 12 weeks of age. The number of mice per group is shown and results are presented as mean and standard error of the mean. * *p* < −0.05; ** *p* < −0.01; **** *p* < −0.0001. PPi: inorganic pyrophosphate.

The remodeling of the ectonucleotidase landscape also suggests long-term compensatory effects could result from ABCC6 deficiency. Genetic compensations ensure the survival and fitness of organisms in spite of genetic perturbations, which is referred to as genetic robustness [231]. It is proposed that compensatory gene networks are established during embryonic development, enabling the organism to adapt to abnormal genetic variations. Recent studies investigating the effects of variation in maternal diets of *Abcc6*^−/−^ mice during gestation revealed the influence of minerals, including PPi, on ectopic mineralization in the offspring [89,156]. Some of the results could be construed as possible genetic compensations. However, we have obtained more direct experimental evidence of potential compensatory mechanisms when investigating the impact of dietary PPi on dystrophic cardiac calcification (DCC) susceptibility in *Abcc6*^−/−^ mice. Interestingly, mice fed a diet rich in PPi exhibited significant inhibition of calcification despite very low PPi bioavailability [133]. Contrary to expectations, reverting to a low PPi diet in adult *Abcc6*^−/−^ animals not only failed to restore the DCC susceptibility of knockout mice but instead led to further calcification inhibition (2/3 additional decrease, Figure 5). This effect was reproducible up to 4 weeks following the dietary change and was consistent between the two existing *Abcc6^tm1Aabb^* and *Abcc6^tm1jfk^* mouse models [30,31]. Moreover, a new generation of mice bred and born on the low PPi diet regained a little more than half of the expected calcification susceptibility (Figure 5). Only the highly modified acceleration diet [232] was able to produce some normalization of the calcification susceptibility after a full month of treatment. These results are the strongest evidence to date for possible compensatory mechanisms with some possible heritable components perhaps in the form of epigenetic changes. Assessing transcriptomic and proteomic profiles as well as the epigenetic landscape in such animal models would provide additional insights into the range of pathophysiologic consequences of ABCC6 and PPi deficiencies.

## 9. Pending Questions and Important Future Research Directions

There are of course many questions remaining regarding the molecular and physiological aspects of PXE and GACI. Below are some of the interrogations we deem most essential.

Despite the effective role of PPi in the prevention of calcification [11,13,89,90,133,210,233,234,235,236,237,238], plasma PPi is not the main determinant of ectopic calcification severity [19,239]. Therefore, establishing methods to determine local concentrations and the exact in situ molecular determinants seems essential to fully understand PXE, GACI and other pathological calcifications such as Randall’s plaque and kidney stones, as well as in arterial calcification resulting from liver failure [240,241,242].

Aside from its well-accepted promotion of ATP efflux, PPi and adenosine generation [11,13,243], ABCC6 also influences extracellular nucleotide metabolism in central and peripheral tissues [20] and cells ([15] and Figure 4), and its deficiency creates a disruptive environment not only conducive to ectopic calcification but likely to other pathogenic processes such as vascular abnormalities [56,97], inflammation [159,160,161], atherosclerosis and dyslipidemia [54]. Further characterization of the impact of ABCC6 deficiency on extracellular nucleotide metabolism could help better understand the precise molecular underpinnings of PXE and GACI and define novel and/or complementary approaches to treating these diseases more effectively (Table 3). 

The precise molecular mechanism(s) by which ABCC6 function leads to ATP efflux [13] also remains a mystery. Initial in vitro vesicular experiments provided the evidence that ABCC6 was an authentic ABC transporter capable of effluxing organic anions in an ATP-dependent fashion such as BQ-123 cyclopentapeptide, leukotriene C4 (LTC4), N-ethylmaleimide S-glutathione (NEM-GS) [42] and S-(2,4-dinitrophenyl) glutathione [115]. However, none of the substrates seem relevant to calcification. For a time ABCC6 was thought to transport vitamin K [119] or adenosine [127], but these suggestions were quickly disproven by physiological experiments and direct transport assays [92,121,122,131]. ABCC6 function leads to ATP efflux and its physiological function with respect to calcification is somewhat understood in hepatocytes. However, ABCC6 is present is several other cell and tissues [105,106,209], so does ABCC6 exert the same physiological function in other cell types?

The approximate 2 to 1 female–male ratio [55] in PXE patients has never been specifically studied or explained. Moreover, what clinical criteria should be used to reliably evaluate and quantify treatment outcomes for PXE patients? This question is constantly debated at PXE conferences, but no solid answer has been proposed yet. This is due to the complexity of the PXE phenotype as multiple organ systems (skin, eyes, cardiovascular, renal. etc.) are affected with various degrees of distribution and severity, and considerable inter- and intra-familial variability [167,251,252]. Indeed, identical mutations can cause the relatively mild PXE or severe GACI [18] in humans but also in mice [85]. The lack of genotype/phenotype correlation only compounds the issue. The presence of modifier genes that could be modulating calcification in PXE has been investigated in human and mice, but their predictive values for clinical applications are still not clear [163,164,165,166,167,168,169]. The identification of such genes/proteins would give precious insights into the molecular partners of ABCC6.

## 10. Conclusions

**Figure 6:**
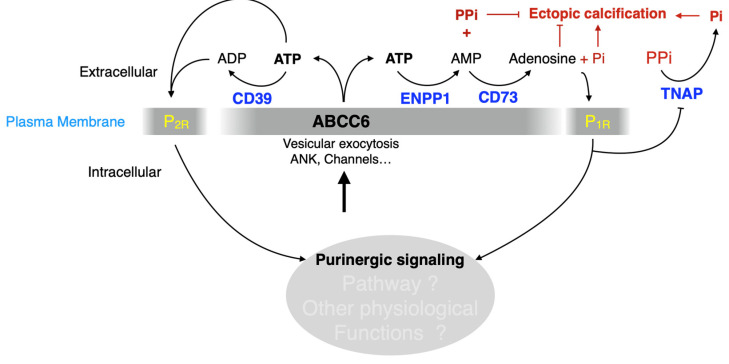
ABCC6 deficiency likely modulates purinergic signaling causing calcification and other pathophysiological dysfunctions. ABCC6 contributes the cellular efflux of ATP from liver and other tissues/cells. This ATP is converted to AMP and PPi by ENPP1 activity, which contributes to calcification inhibition. We also suggest that ATP is also hydrolyzed to ADP by CD39 and signals towards P2 receptors (P2_R_). AMP is further dephosphorylated by CD73 (*NT5E*) to adenosine, which signals via the P1 receptors (P1_R_) to not only inhibit TNAP synthesis but likely also affect other molecular pathways. ABCC6 deficiency leads to a significant decrease in plasma purines and changes in ectonucleotidase expression both in vivo and in vitro [15,20,54,88,173]. This literature along with original results presented in this review (cf. Figure 3, Figure 4 and Figure 5) strongly suggest that the lack of ABCC6 alters purinergic signaling in a variety of tissues and is the cause of pathologic manifestations other than calcification in PXE.

ATP efflux is the currently accepted molecular function of ABCC6. ATP crosses the cell membrane via several tightly-regulated mechanisms and modulates a plethora of cellular functions under various pathophysiological conditions through P2 receptor activation [192]. The hydrolysis of ATP generates adenosine, which exerts wide vasculoprotective and anti-inflammatory effects through P1. Among the molecular candidates for ATP cellular release pathways, there are ATP-binding cassette (ABC) proteins including ABCB1, ABCC7 and now ABCC6. The molecular mechanisms underlying ABCC6-dependent ATP release remain largely unknown. Recent and compelling recent evidence (from our work and others) show that ABCC6 deficiency impacts distal nucleotide metabolizing enzymes and it is logical to assume downstream influence on P1 vs. P2 receptors, whose action on cardiovascular homeostasis and other physiological functions are well documented (Figure 6).

PXE and GACI are rare diseases that have been extensively studied over the last 2 decades and from which much has been learned. The gained knowledge is progressively being extended and applied to other diseases with ectopic calcification, for example the development Randall’s plaque and kidney stones, and arterial calcification resulting from kidney or liver failure as well as systemic sclerosis [235,240,241,242,253]. We have described in this review a series of well-documented evidence reporting the dysregulation of purinergic signaling and related gene expression as a result of ABCC6 deficiency. This aspect of the pathobiology of PXE/GACI deserves a lot more attention as this undoubtedly contributes to ectopic calcification and most likely other manifestations of the diseases. Understanding the full molecular ramification of ABCC6 deficiency represents an opportunity to better treat PXE/GACI and also ectopic calcification when it manifests as a co-morbidity in much more common pathologies such as diabetes, kidney failure, atherosclerosis or simply aging.

## Figures and Tables

**Table 1:** Mutations associated to human diseases and mouse calcifying phenotype. OMIM (Online Mendelian Inheritance in Man^®^ reference); NR, not reported.

Protein	Human Pathology	OMIM	Function	Target Gene	Vascular Calcification	Vibrissae Calcification	Bone Defect	Refs.
***ABCC6***	Pseudoxanthoma elasticum	264800	ABC transporter—cellular nucleotide/ATP release	*Abcc6^−/−^*	Moderate progressive	Yes progressive	Trabecular bone loss with ageing	[30,31,146]
***ANKH***	Craniometaphyseal dysplasia, autosomal	118600/123000	Cellular PPi/ATP release	*Ank^−/−^*	High early lethality	NR	Yes	[141,144]
***ENPP1***	General Arterial Calcification of Infancy	208000	Ectonucleotide pyrophosphatase phosphodiestherase: ATP → PPi	*Enpp1^−/−^*	Med.	Yes	Yes	[147,148,149]
***NT5E***	Calcification of joints and Arteries	211800	Ecto 5’-nucleotidase—Adenosine generation	*Nt5e^−/−^*	ND	Yes	NR	[19,86]
***PANNEXIN-1***	NR	608420	Membrane anionic channel, nucleotide permeation	*Panx1^−/−^*	NR	No (unpublished observation)	Major	NR
***NTPDase1***	NR	601752	Ectonucleoside triphosphate diphosphohydrolase-1 ATP/ADP → AMP = Pi	*Entpd1^−/−^*	NR	No (unpublished observation)	NR	NR
***TNAP***	Hypophosphatasia	146300	Tissue nonspecific alkaline phosphatase ATP ADP AMP PPi → ADO + Pi	*Alpl^−/−^*	NR	NR	Yes	[150]
***MGP***	Keutel syndrome	245150	Gamma carboxylation	*Mgp^−/−^*	Light	NR	NR	[151]
***OPG***	Juvenil Paget Disease/Hyperostosis corticalis deformans juvenilis	239000	Decoy receptor for RANKL	*Opg^−/−^*	Med/high subintimal	NR	NR	[152]
***SPP1***	NR	166490	Osteopontin	*Opn^−/−^*		NR	NR	[153]
***KLOTHO***	Tumoral calcinosis, hyperphosphatemic	211900	Hormone	*Kl^−/−^*	Med/high	NR	NR	[154]

On a related note, we have shown in a recent study that PXE patients and *Abcc6^−/−^* mice have a highly significant 2-fold decrease in plasma ATP and ADP levels [20]. The origin of this deficit is not clear and needs to be investigated.

**Table 2:** Altered purine metabolism and transport in ABCC6 deficiency. Enzymes were evaluated through Protein (P) expression, Transcription (T) or Activity (A). ND means not determined.

Reference	Cell/Tissue	Protocol	NPP1/*ENPP1*	CD73/*NT5e*	TNAP/*ALPL*	CD39/*ENTPD1*	ANKH	PIT/*SLC20A1*
[182]	PXE skin fibroblasts	Calcifying medium	**T+ P+** (20 days) **T− (8 h)**	ND	**A+** (20 days)	ND	**T= P=**	ND
[163]	PXE skin fibroblasts	Calcifying medium	**T− A−**	ND	**T+**	ND	**T=**	**T+**
[163]	PXE skin fibroblasts	phosphate medium	**T+ A+**	**T= A−**	**T+ A+**	ND	ND	ND
[162]	*Abcc6^−/−^* (males) skin fibroblast	Ageing	**P=**	ND	**P= A+**	ND	**P−**	ND
[20]	PXE serum	no	**A=**	**A+**	**A=** (*p* = 0.1)	**A=**	NA	ND
[183]	PXE serum	no	**A=**	ND	**A+**	ND	ND	ND
[20]	*Abcc6^−/−^* (females) serum	Ageing	**A−**	**A+**	**A=**	**A=**	ND	ND
[20]	*Abcc6^−/−^* (females) thoracic aorta	Ageing	**T+**	**T+**	**T=**	**T+**	**T+**	**T+**
**Le Saux unpublished**	*Abcc6^−/−^ (males) Lymphocytes*	no	**T=**	**T+**	**T−**	ND	**T−**	ND
**Brampton et al. unpublished**	*Abcc6^−/−^ (males) Macrophages*	no	**T+**	**T+**	**T=**	ND	**T+**	ND
[20]	*Abcc6^−/−^* (females) liver	Ageing	**T=**	**T−**	**T=**	**T+**	**T=**	**T=**
**Brampton et al. unpublished**	*Abcc6^−/−^* (males) liver	no	**T−**	**T−**	ND	ND	ND	ND
[15]	HepG2 cells	shRNA ABCC6	**T=**	**T− P−**	**T+ P+ A+**	ND	ND	ND

**Table 3:** Various molecules directly or indirectly linked to purine metabolism and ectopic calcification as potential pharmaceutical targets.

Treatment	Patent	Clinical Trial	Indication	Outcome in PXE Diease	Therapeutically Action	References
PPi	P32885NL00/RKI	NCT04868578	No	ongoing	Interference with HA crystal growth	[89,90]
Bisphosphonate (Etidronate)	NR	NL4956 NCT05832580	Menopause	reduction in arterial calcification and subretinal neovascularization	Interference with HA crystal growth	[21,244,245]
INZ-701	NR	NCT06046820NCT05030831	GACI/PXE	ongoing	Recombinant ENPP1 enzyme - PPi generation	[135,246]
TNAP Inhibitor (Lansoprazole)	WO2016054056A1	NCT04660461		Increase plasma PPi in PXE patients	Prevent PPi hydrolysis and Pi accumulation	[19,171,247]
ADO	US20130109645A1	No	Coronary dilation for stress echocardiography	?	Reduction in TNAP expression, PPi hydrolysis and Pi generation	[14,127]
ARL67156	No	No	No	?	Inhibition of ectonucleotidase with ARL67156 prevents the development of calcific aortic valve disease in warfarin-treated rats	[248]
ATP	No	No	No	?	Replete deficient ATP levels prevents vascular calcification and extends longevity in a mouse model of Hutchinson-Gilford progeria syndrome	[249]
Apyrase	University of Michigan 61707228	No	No	?	Apyrase (CD39-like ATPDase) accelerate adenosine formation and prevents heterotypic ossification through decreasing MSC ostegenic differenciation	[250]
Methotrexate	No	No	Rheumatoid arthritis, Inflammatory Bowel Disease, Autoimmune diseases	?	Enhanced CD73-dependent adenosine production - prevension of inflammation	[175]
Dypiridamol	No	No	Platelet inhibition, coronary dilation for stress echocardiography	?	Anti-platelet drug – Increase extracellular adenosine through inhibition of cellular uptake by nucleside transporters ENT1 and ENT2	[201]

## Data Availability

No large datasets were generated or analyzed during the current study.
